# Glycyrrhizin mitigates radiation‐induced acute lung injury by inhibiting the HMGB1/TLR4 signalling pathway

**DOI:** 10.1111/jcmm.14703

**Published:** 2019-10-27

**Authors:** Lei Zheng, Qian Zhu, Cheng Xu, Min Li, Huan Li, Pei‐Qiang Yi, Fei‐Fei Xu, Lu Cao, Jia‐Yi Chen

**Affiliations:** ^1^ Department of Radiation Oncology Ruijin Hospital Shanghai Jiaotong University School of Medicine Shanghai China

**Keywords:** glycyrrhizin, HMGB1, radiation‐induced lung injury (RILI), RAGE, TLR4

## Abstract

Radiation‐induced lung injury (RILI) is the major complication of thoracic radiation therapy, and no effective treatment is available. This study explored the role of high‐mobility group box 1 (HMGB1) in acute RILI and the therapeutic effect of glycyrrhizin, an inhibitor of HMGB1, on RILI. C57BL/6 mice received a 20 Gy dose of X‐ray radiation to the whole thorax with or without administration of glycyrrhizin. Severe lung inflammation was present 12 weeks after irradiation, although only a mild change was noted at 2 weeks and could be alleviated by administration of glycyrrhizin. Glycyrrhizin decreased the plasma concentrations of HMGB1 and sRAGE as well as TNF‐α, IL‐1β and IL‐6 levels in the bronchoalveolar lavage fluid (BALF). The expression of RAGE was decreased while that of TLR4 was significantly increased at 12 weeks, but not 2 weeks, after irradiation in mouse lung tissue. In vitro, the expression of TLR4 increased in RAW 264.7 cells after conditioning with the supernatant from the irradiated MLE‐12 cells containing HMGB1 but showed no change when conditioned medium without HMGB1 was used. However, conditioned culture had no effect on RAGE expression in RAW 264.7 cells. Glycyrrhizin also inhibited the related downstream transcription factors of HMGB/TLR4, such as NF‐κB, JNK and ERK1/2, in lung tissue and RAW 264.7 cells when TLR4 was activated. In conclusion, the HMGB1/TLR4 pathway mediates RILI and can be mitigated by glycyrrhizin.

## 1 INTRODUCTION

Despite the progress in modern radiotherapy techniques, radiation‐induced lung injury (RILI) remains the main complication of radiotherapy for thoracic malignancies.^1^ RILI is characterized by a relatively long latent stage, subsequent acute inflammation and a final fibrosis stage.^2^ The latent stage has no obvious pathophysiologic changes, while the inflammatory stage is accompanied by epithelial cell collapse, increased lung capillary permeability and inflammatory cell infiltration. In addition, acute inflammation sometimes causes acute respiratory distress syndrome (ARDS) and death.^3^ To date, no drugs for RILI have been approved by the Food and Drug Administration (FDA), and the exact mechanism by which radiation induces inflammatory responses in the lung remains unclear. Oxidative stress, hypoxia, immunocytes and cytokines all play roles in the development of radiation‐induced pneumonitis.^4^ Recent reports demonstrated that innate immunity triggered by radiation acted as an immune modulator, causing radiation‐induced damage in normal tissue.^5^, ^6^

High‐mobility group box 1 (HMGB1) is a DNA molecular chaperone that is constitutively expressed in the cell nucleus and serves as a damage‐associated molecular pattern (DAMP) when it is released into the extracellular space.^7^ DAMPs, similar to pathogen‐associated molecular patterns (PAMPs), can activate the immune system by binding to pattern recognition receptors (PRRs).^8^ HMGB1 was first identified as a late mediator in a sepsis model^9^ and is now thought to play a vital role in many diseases.^10^ An increasing number of reports have confirmed the functions of HMGB1 in infectious and sterile inflammation, and many therapies targeting HMGB1 have been developed.^11^, ^12^ Some studies reported that radiation could lead to HMGB1 translocation and release, but its role in RILI has not been explicitly revealed.^13^, ^14^

Glycyrrhizin (GL), a glycoconjugated triterpene extracted from the roots and rhizomes of liquorice, has been widely used in Japan and China as a hepatic protector and anti‐inflammatory agent for decades. GL has been shown to be a natural inhibitor of HMGB1 by binding to the HMGB1 protein directly and inhibiting its biological activity.^15^ In vital organs, GL has a protective role against porcine endotoxaemia by reducing the expression of HMGB1 and pro‐inflammatory cytokines.^16^ GL decreased serum levels of HMGB1 in a rat sepsis model, protected against carrageenan‐, benzo(a)pyrene‐, and lipopolysaccharide‐induced lung injury and ischaemia‐reperfusion lung injury in vivo, and reduced mortality as a result.^17^ Although few reports are available on the radioprotective effect of GL against RILI,^18^, ^19^ they only observe the protective effect of GL against radiation and do not explore its mechanism as an HMGB1 inhibitor. Whether HMGB1 participates in the process of RILI and GL can mitigate RILI by inhibiting HMGB1 has not been reported until now.

To explore the role of HMGB1 in the development of RILI and the radioprotective mechanism of GL, we performed studies and tried to find whether GL can mitigate RILI by inhibiting the HMGB1/TLR4 signalling pathway.

## 2 MATERIALS AND METHODS

### 2.1 Animal model and irradiation

Adult female C57BL/6 mice (8 weeks of age, 17‐19 g) were obtained from the Experimental Animal Center of Rui Jin Hospital in Shanghai, China. All animal experimental procedures were approved by the Animal Ethics Committee of Rui Jin Hospital and were performed in accordance with the Guide for The Care and Use of Laboratory Animals published by the National Institutes of Health. The mice were maintained under a 12‐h light/dark cycle with standard food pellets and water available ad libitum. Before irradiation, the mice were randomly divided into four groups: normal control (C group); GL without irradiation (CG, Control + GL group); irradiation only (R group); and irradiation plus GL (RG, Radiation + GL group).

After anaesthetization by intraperitoneal administration of 50 mg/kg 1% sodium pentobarbital, each mouse was fixed on an organic glass pedestal with the body fully stretched. The whole thorax received a single 20 Gy photon dose (6 MeV, dose rate 300 cGy/min, source surface distance of 1 m; Elekta Precise, Stockholm, Sweden). The irradiation field was 40 × 2 cm, according to the breadth of the pedestal and the lung size of 8‐week‐old mice. Before irradiation, the irradiated area of each mouse was verified under X‐ray simulator to ensure that the head and abdomen were shielded. GL (Selleck, Houston, USA; 10 mg/kg in 2% DMSO/PEG 400) was administered intraperitoneally (*i.p*.) 1 hour before irradiation and every day until week 4 and then three times per week until week 12.

### 2.2 Histological evaluation

After opening the chest, the lungs were perfused with PBS according to the standard protocol, and the removed tissues were fixed in 4% paraformaldehyde and embedded in paraffin. Sections (5 µm) were stained with haematoxylin and eosin (H&E) and evaluated by two experienced pathologists who were blinded to the experimental treatment groups. The pneumonia scores were assessed by evaluating the thickness of the alveolar septa and infiltrated inflammatory cells in the alveolar and interstitial space, as previously reported.^20^, ^21^

### 2.3 Bronchoalveolar lavage fluid analysis

The mice were killed, the trachea was fully exposed, and a small plastic catheter was inserted into the trachea. After irrigation with 0.5‐1 mL of PBS, bilateral BALF was collected, and the process was repeated 2‐3 times until the total BALF volume reached approximately 1 mL. The collected BALF was centrifuged at 1500*g* for 15 minutes, and the supernatant was frozen at −80°C for ELISA. After removing the supernatant, the pellet was resuspended in 0.5 mL of PBS for cell counting and then stained with the Diff‐Quick method with cytospins for differential cell counts for statistical analysis.

### 2.4 Cell line and irradiation

The mouse alveolar epithelial cell line (MLE‐12) and mouse monocyte cell line (RAW 264.7) were purchased from ATCC (Manassas). MLE‐12 cells were cultured with F12/DMEM supplemented with 10% foetal bovine serum (FBS; Gibco) in a 6‐cm culture dish; the medium was replaced with serum‐free medium 2 hours before irradiation. Cell irradiation was also performed under Linac with an 8 Gy dosage. RAW 264.7 cells were cultured with DMEM with 10% FBS. All cells were cultured at 37°C in a humidified atmosphere of 95% and 5% CO_2_.

### 2.5 Migration assay

Cell migration assays were performed using a transwell migration chamber (Corning). MLE‐12 cells were seeded in a 24‐well cell culture plate with 10% F12/DMEM with or without HMGB1 peptide‐free lipopolysaccharide immediately after irradiation; transwell inserts were suspended in the individual wells. A total of 1 × 10^5^ RAW 264.7 cells were seeded in the upper chamber of 8‐µm porous transwell inserts without Matrigel in serum‐free DMEM with or without GL. After co‐culturing for 24 and 48 hours, the upper chambers were removed and fixed with 4% paraformaldehyde for 20 minutes at room temperature. Then, cells were stained with crystal violet, and after washing with PBS, non‐migrated cells were removed from the transwell chambers with a cotton swab. We counted the number of migrated cells from five different microscope fields for statistical analysis.

### 2.6 Conditioned culture

To mimic the process of RILI in vitro, we applied the supernatants from irradiated MLE‐12 cells as conditioned medium and observed the effect on RAW 264.7 cells, which represent infiltrated immunocytes in vivo. HMGB1 expression was detected in the irradiated supernatants by ELISA 24 and 48 hours after replacing the original medium of RAW 264.7 cells when the confluence was approximately 70% and the cells were maintained in culture for an additional 24 hours.

### 2.7 Western blot analysis

Tissue samples and cells were homogenized with RIPA lysis buffer (Beyotime Biotech, Shanghai, China). Protein concentrations were determined by using a BCA protein assay kit. The proteins were separated on a 12% SDS‐polyacrylamide gel and transferred to a PVDF membrane. After blocking with 5% bovine serum albumin (BSA) in TBS, the membrane was incubated with primary antibody at 4°C overnight. The secondary antibody was conjugated to horseradish peroxidase and incubated at room temperature for 1 hour. Immunoreactivity was detected with an enhanced chemiluminescence kit (Beyotime Biotech) by a ChemiDoc™ MP System (Bio‐Rad). The following antibodies were used: HMGB1 (1:5000, Abcam), RAGE (1:1000, Abcam), RAGE (1:200, Santa Cruz), TLR4 (1:1000, Protein Tech), TLR4 (1:200, Santa Cruz) and β‐actin (1:1000, Abcam); pNF‐κB/NF‐κB, pJNK/JNK and pERK/ERK were all purchased from Cell Signaling Technology and incubated at a dilution of 1:1000.

### 2.8 RNA isolation and real‐time PCR

Total RNA was extracted from frozen lung tissue using TRIzol Reagent (Invitrogen) according to the manufacturer's instructions. Then, the total RNA was reverse‐transcribed into CDNA with a PrimeScript™ RT reagent kit with gDNA Eraser (Takara, Japan). Real‐time PCR was performed by monitoring the intensity of fluorescence in real time using SYBR Green dye (Takara, Japan) with a StepOne™ Real‐time PCR machine (Applied Biosystems). The PCR primer pairs used were as follows: β‐actin, forward, 5′‐ATGACAACTTTGGCATTGTG‐3′ and reverse, 5′‐CATACTTGGCAGGTTTCTCC‐ 3′; HMGB1, forward, 5′‐TTGTGCGAAAAGAAGTGC AG‐3′ and reverse, 5′‐TACAAACACAGCCTCCCACA‐ 3′; RAGE, forward, 5′‐TGGCTCGAATCCTCCCCAAT‐3′ and reverse, 5′‐CCTCCTTCCCTCGCCTGTTA‐3′; and TLR4, forward, 5′‐GAGCCGGAAGGTTATTGTGGTAGTG‐3′ and reverse, 5′‐TCAAGGACAATGAAGATGATGCCAGAG‐3′.

### 2.9 ELISA

The BALF concentrations of TNF‐α, IL‐1β and IL‐6 were measured using ELISA kits (Beyotime Biotech), and the plasma levels of HMGB1 and sRAGE were detected by an IBL ELISA kit (Japan) and R&D ELISA kit (American), respectively, according to the manufacturer's directions.

### 2.10 Statistical analyses

Data are represented as the mean ± SEM from at least three independent experiments. All variables were analysed by one‐way ANOVA or Student's *t* tests. Statistical significance was defined as *P* < .05.

## 3 RESULTS

### 3.1 Radiation caused pulmonary inflammation, and GL mitigated acute RILI

**Figure 1:**
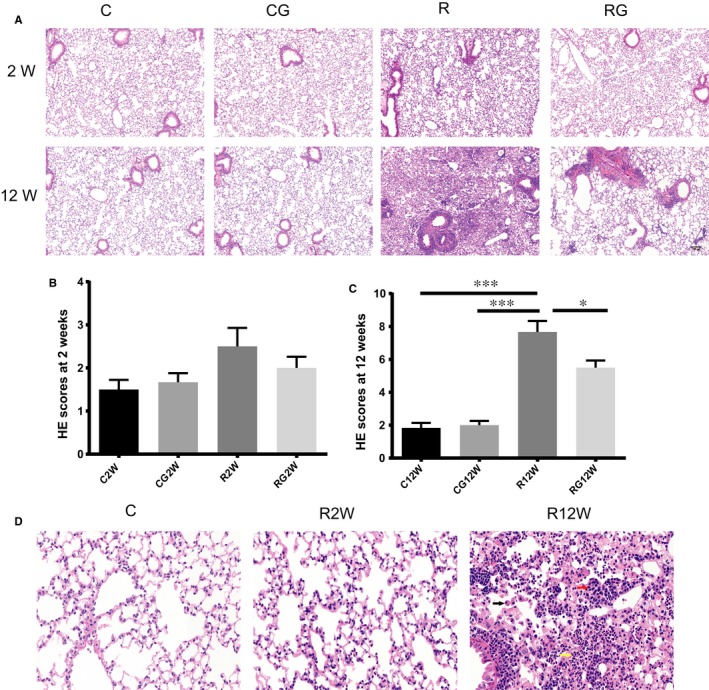
GL alleviates lung inflammation after radiation. A, Representative images of H&E staining at 2 and 12 wk, Scale bar = 100 µm. B,C, Lung tissue inflammation scores at 2 and 12 wk after irradiation (n = 6, *P < .5, **P < .01, ***P < .001). D, Magnified images of H&E staining of no radiation and 2/12 weeks after irradiation. Main infiltrating cell types in lung tissue after 12 weeks are marked by arrows: black arrow, macrophages; red arrow, lymphocytes; yellow arrow, neutrophils. Scale bar = 50 µm

Radiation caused obvious pulmonary inflammation at 12 weeks, indicated by macrophage, lymphocyte and neutrophil infiltration in the alveoli and interstitium via histopathology. For mice treated with GL, infiltrated immunocytes in lung tissue were decreased significantly at 12 weeks after irradiation, as shown in Figure 1. The immediately injury induced by radiation was not reflected by pathological changes, only a moderate inflammatory response at 2 weeks after irradiation demonstrating a latent period.

### 3.2 GL inhibited immunocyte infiltration into the BALF

**Figure 2:**
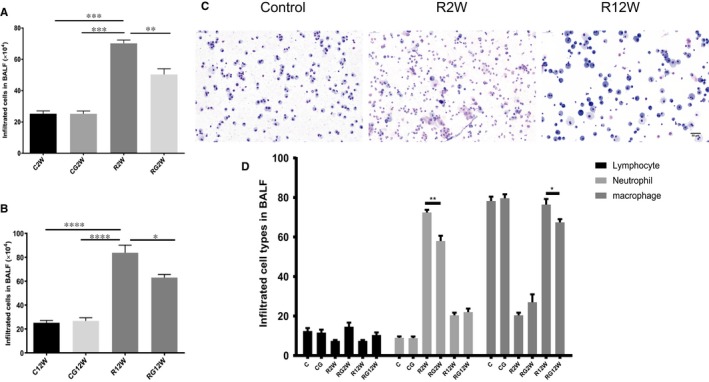
Infiltrating cells in the BALF at 2 and 12 wk. A,B, Total numbers of infiltrating cells at 2 and 12 wk in the BALF. C, Representative Diff‐Quick staining images of cells in the BALF of no radiation and at 2 and 12 wk after irradiation. Scale bar = 50 µm. D, Statistical analysis results of three main cell types in the BALF after irradiation. (*P < .5, **P < .01, ***P < .001)

Although lung inflammation at 2 weeks was mild, infiltrated cells increased noticeably in the BALF. GL decreased the total number of infiltrating immunocytes in the BALF and changed the distribution of inflammatory cell types. Figure 2 shows that the main cells in the BALF from mice receiving no radiation were small alveolar macrophages. Neutrophils were the main cells in the BALF at 2 weeks; however, at 12 weeks, the primary cells were macrophages, and most of them had polarized, exhibiting a larger morphology. GL significantly decreased the number of neutrophils and macrophages in the BALF at 2 and 12 weeks after irradiation, respectively.

### 3.3 GL decreased the levels of cytokines in the BALF

**Figure 3:**
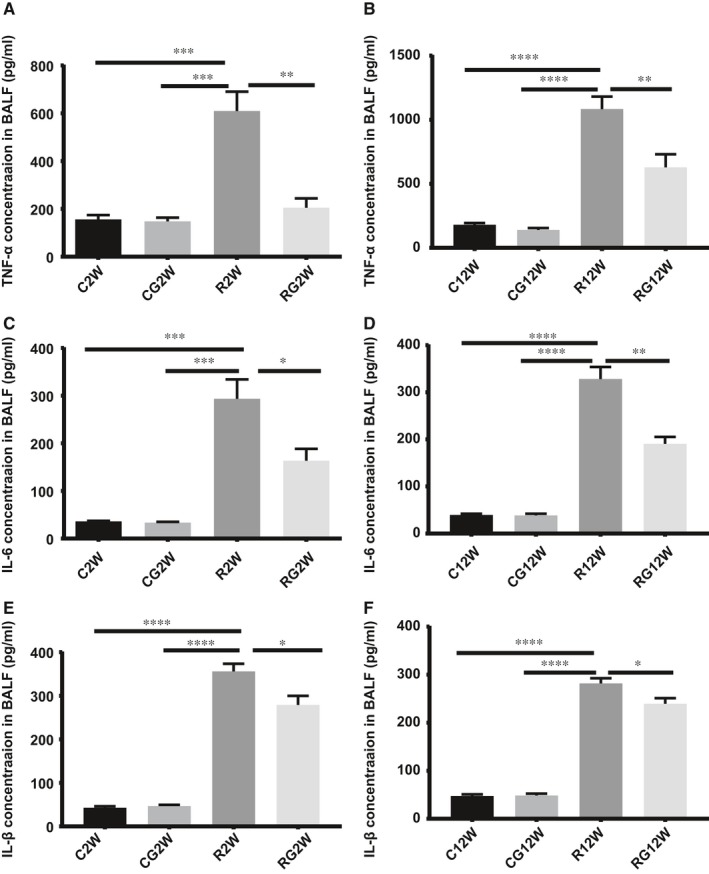
Cytokine concentrations in the BALF. TNF‐α, IL‐6 and IL‐1β concentrations in the BALF were measured by ELISA. A,C,E, 2 wk after irradiation; B, D, F, 12 wk after irradiation. (*P < .5, **P < .01, ***P < .001)

At 2 and 12 weeks after irradiation, TNF‐α, IL‐1β and IL‐6 in the BALF increased significantly compared to those without radiation exposure, and GL inhibited the release of these pro‐inflammatory cytokines after irradiation (Figure 3).

### 3.4 GL inhibited HMGB1 synthesis and release in vivo

**Figure 4:**
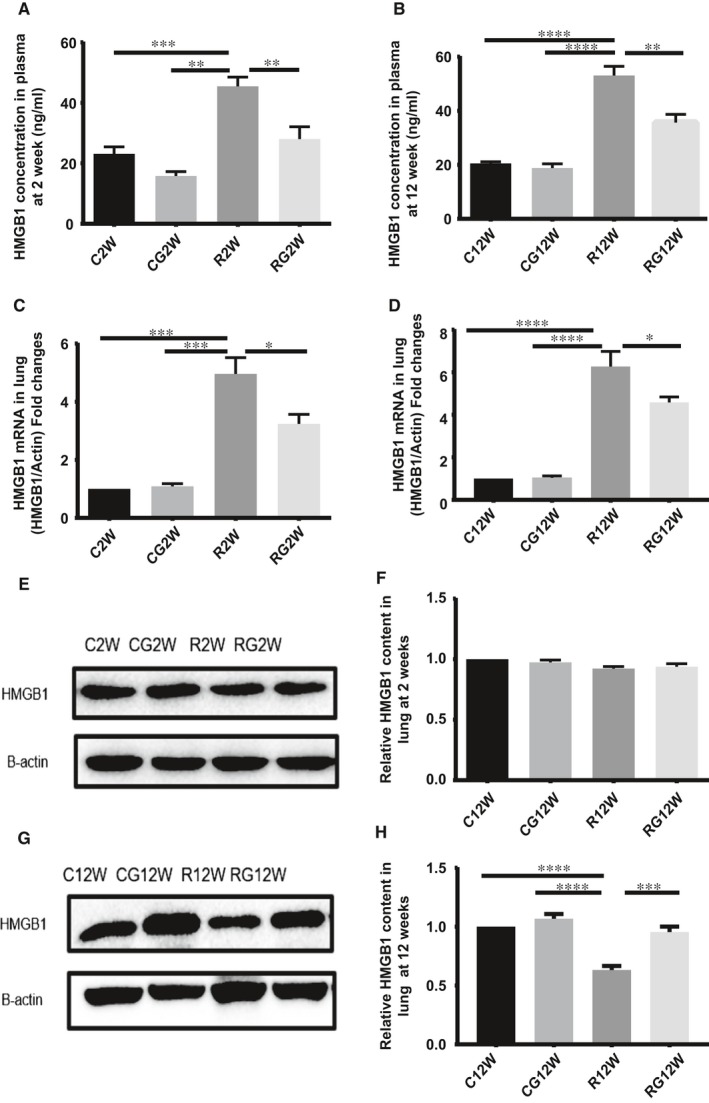
Glycyrrhizin inhibits HMGB1 synthesis and release. A,B, Plasma level of HMGB1 detected by ELISA at 2 and 12 wk after irradiation. C,D, Relative mRNA expression of HMGB1 in the lung tissue after irradiation. E,G, Western blotting analysis of HMGB1 in the lung tissues at 2 and 12 wk after irradiation. F,H, Relative qualification of HMGB1 content in the lung tissue after irradiation. (*P < .5, **P < .01, ***P < .001)

At the latent and inflammation stages of RILI, both mRNA in lung tissue and plasma levels of HMGB1 increased significantly, while the change in HMGB1 protein in the lung tissue was inconsistent, with a mild change at 2 weeks after irradiation but a significant decrease at 12 weeks. Glycyrrhizin can inhibit HMGB1 synthesis and release both in the latent and inflammation stages of RILI (Figure 4).

### 3.5 Expression of RAGE and TLR4 in lung tissue after irradiation

**Figure 5:**
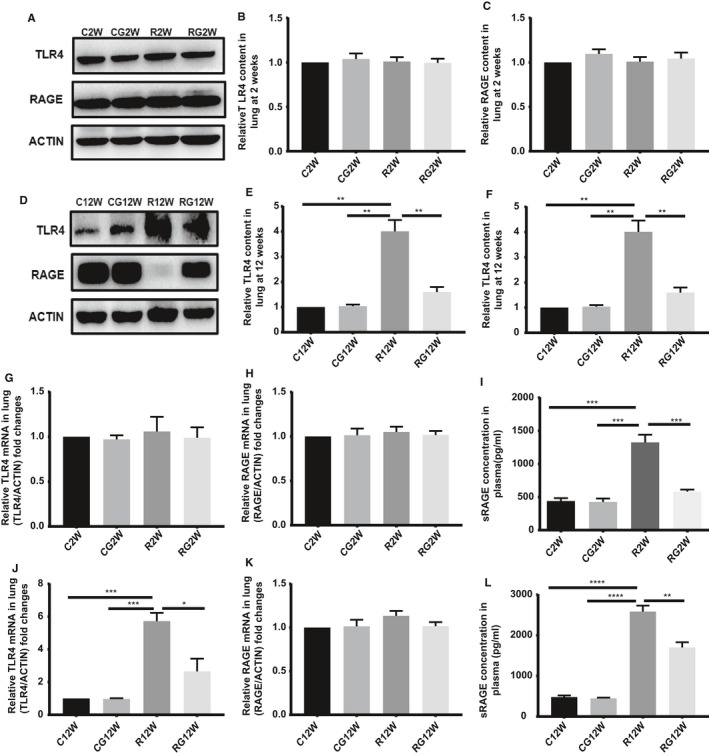
Expression of HMGB1 receptors in the lung tissue during different stages after radiation. A,D, Western blotting analysis of TLR4 and RAGE in the lung tissues at 2 and 12 wk after irradiation. B,C,E,F, Relative qualification of RAGE and TLR4 content in the lung tissue in 2 and 12 wk after irradiation. G,H,J,K, Relative mRNA expression of RAGE and TLR4 in the lung tissue at 2 and 12 wk after irradiation. I,L, ELISA results of sRAGE in plasma during different stages after irradiation. (*P < .5, **P < .01, ***P < .001)

At the latent stage of RILI, both RAGE and TLR4 expression in the lung tissue changed slightly, but during the inflammation stage, RAGE levels decreased while those of TLR4 increased significantly. Radiation caused increased TLR4 mRNA expression at the inflammation stage but had little effect on RAGE mRNA at both the latent and inflammation stages of RILI. In contrast to the change in RAGE in the lung tissue, radiation caused the soluble RAGE in plasma to increase persistently along with the damage to alveolar epithelial cells (Figure 5).

### 3.6 Glycyrrhizin blocked the chemotaxis of HMGB1 in vitro

**Figure 6:**
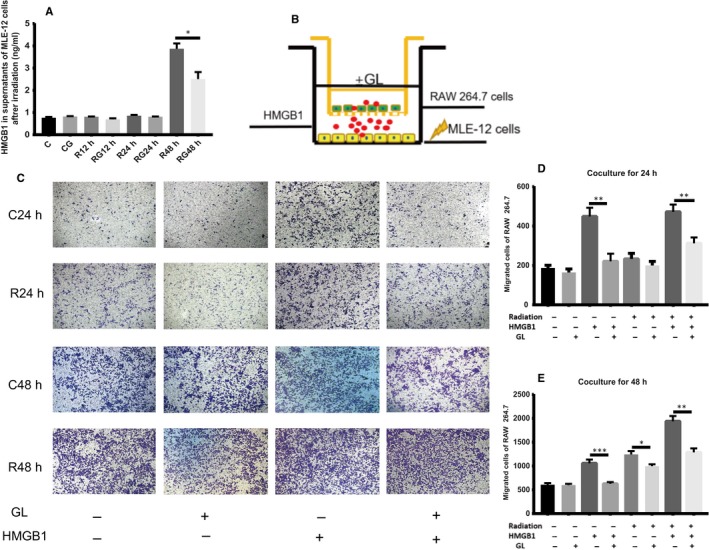
GL blocks the chemotaxis of HMGB1 in vitro. A, HMGB1 in supernatants from MLE‐12 cells after irradiation at different times by ELISA. HMGB1 was increased only at 48 h after irradiation. B, Scheme of the transwell assay. MLE‐12 cells were seeded in the lower compartment and underwent irradiation, while RAW 264.7 cells were seeded in the upper chamber and co‐cultured for 24 and 48 h, respectively. C, Representative images of migrated cells stained with crystal violet after co‐culturing for different times. 100× D,E, Result of migrated cell counts after co‐culturing for 24 and 48 h. (*P < .5, **P < .01, ***P < .001)

To mimic the chemotaxis of irradiated lung tissue to peripheral blood mononuclear cells, we used MLE‐12 and RAW 264.7 cells as alveolar epithelial cells and peripheral monocytes, respectively, in vitro. MLE‐12 cells were irradiated with 8 Gy dosage, and HMGB1 was detected 48 hours after irradiation but not 24 hours. Transwell assays showed that migrating RAW 264.7 cells increased significantly at 48 hours when HMGB1 was released, but not at 24 hours when HMGB1 was still located intracellularly. We also found that the addition of HMGB1 peptide promotes the migration of RAW 264.7 cell in control and radiation groups as well as glycyrrhizin inhibits the migration stimulated by HMGB1 (Figure 6).

### 3.7 GL inhibited the expression of TLR4 in immunocytes after conditioned culture

**Figure 7:**
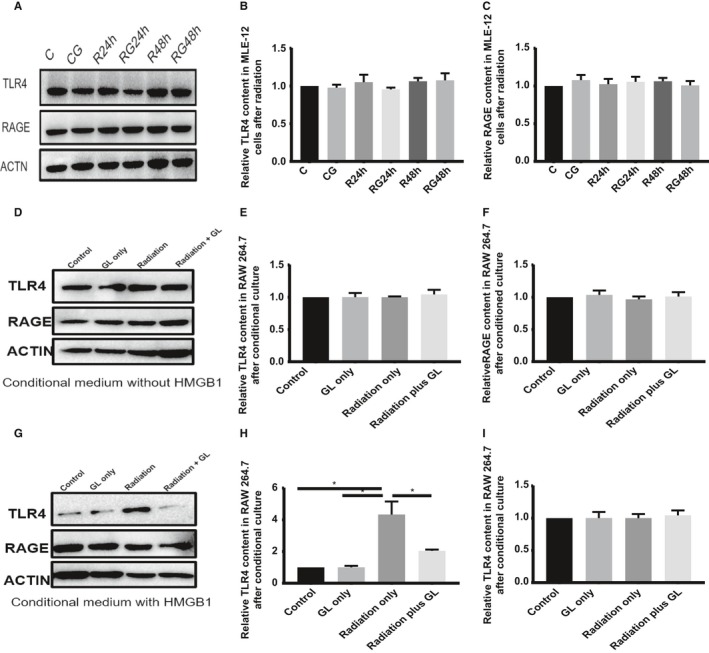
GL inhibits TLR4 expression in immunocytes after conditioned culture. A, Western blotting analysis of TLR4 and RAGE in MLE‐12 cells at 24 and 48 h after irradiation in different groups. B,C, Relative qualification of RAGE and TLR4 content in MLE‐12 cells after irradiation. D,G, Western blotting analysis of TLR4 and RAGE in RAW 264.7 cells after conditioned culture with (48‐h supernatants of MLE‐12 cells after irradiation) or without (24‐h supernatants of MLE‐12 cells after irradiation) released HMGB1. E,H, Relative qualification of RAGE in RAW 264.7 cells after conditioned medium. F,I, Relative qualification of TLR4 in RAW 264.7 cells after conditioned medium

To exclude the interference of different cell types between the inflammation stage and latent stage in vivo, we examined the receptors of HMGB1 in irradiated MLE‐12 cells and RAW 264.7 cells after conditioned culture. Radiation had no direct effect on the expression of RAGE and TLR4 in MLE‐12 cells. The supernatant of the irradiated MLE‐12 cells had a moderate effect on the expression of RAGE in RAW 264.7 cells. However, the expression of TLR4 increased significantly after culture with supernatants of irradiated MLE‐12 cells containing the released HMGB1 at 48 hours post‐irradiation. Glycyrrhizin inhibited the expression of TLR4 in RAW 264.7 cells, which was activated by supernatants from irradiated MLE‐12 cells at 48 hours post‐irradiation (Figure 7).

### 3.8 GL inhibited HMGB1/TLR4 downstream in vivo and in vitro

**Figure 8:**
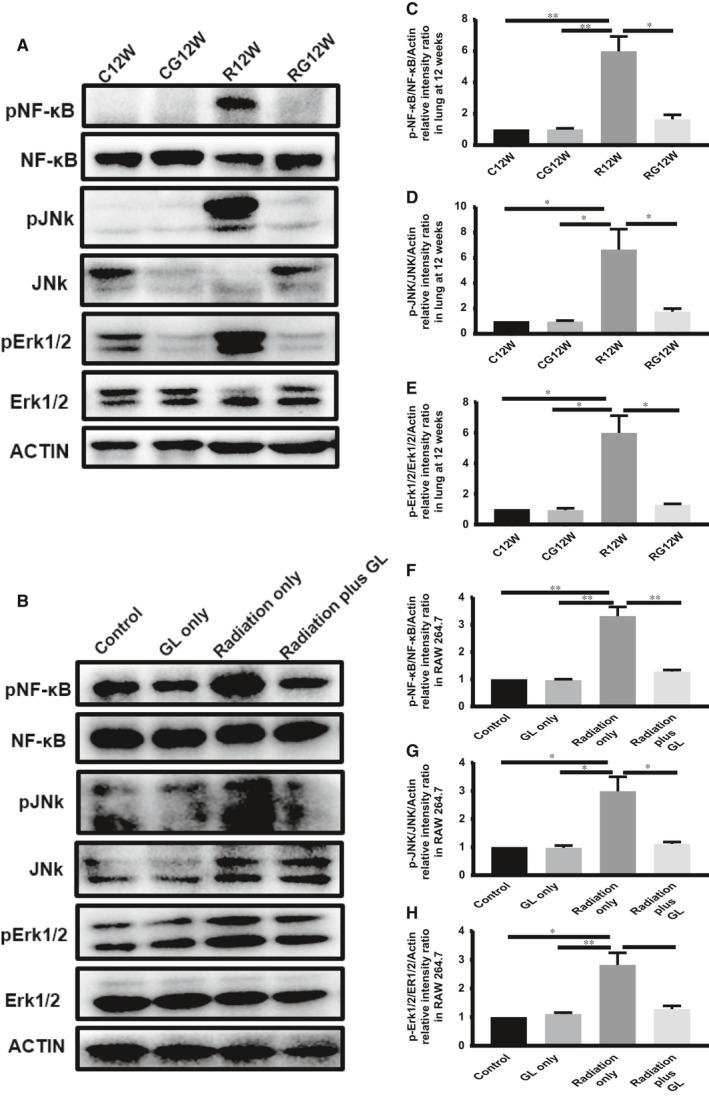
GL inhibits the downstream of HMGB1/TLR4 signalling pathway in vivo and in vitro. A, Western blotting analysis of NF‐κB, JNK and ERK1/2 in the lung tissue at 12 wk after irradiation. C,D,E, Relative qualification of pNF‐κB, pJNK and pErk1/2 in lung tissue at 12 wk. B, Western blotting analysis of NF‐κB, JNK and ERK1/2 in RAW 264.7 cells after conditioned culture. F,G,H, Relative qualification of pNF‐κB, pJNK and pErk1/2 in RAW 264.7 cells after conditioned culture

Many transcription factors are involved in the HMGB1/TLR4 signalling pathway; we examined the expression of NF‐κB, JNK and ERK1/2 in vivo and in vitro when TLR4 was activated. We found that pNF‐κB, pJNK and pERK1/2 increased significantly in lung tissues during the inflammation stage of RILI and in RAW 264.7 cells when cultured with conditioned medium containing HMGB1. Interestingly, glycyrrhizin can block the activation of these downstream transcription factors in vivo and in vitro (Figure 8).

## 4 DISCUSSION

The incidence of clinical RILI is observed in up to 30% of lung cancer patients receiving thoracic irradiation and approximately 10%‐15% in other thoracic malignancy patients.^22^ Although RILI has been known for almost one hundred years, its molecular mechanism is not fully clarified, and no effective therapy is available except for steroid corticoids and antibiotics in case of subsequent infection. HMGB1, a DAMP molecule, has been demonstrated to be involved in infectious and sterile inflammation,^23^ but its role in RILI is unclear. Our study found that HMGB1 plays an important role in mediating RILI and that inhibition by GL can alleviate RILI.

Radiation‐induced lung injury has a relatively long latent stage that exhibits no obvious pathological changes, but many cytokines have already been investigated in vivo and throughout the whole process of RILI.^24^, ^25^ Radiation induces acute lung inflammation that often occurs 3‐12 weeks after irradiation, with monocytes, neutrophils and lymphocytes infiltrating the alveolar interstitium.^24^ Innate and adaptive immunity are known to have functions in radiation‐induced inflammation, but how radiation initiates the immune system is not yet obvious.^26^ Recent studies have identified endogenous DAMPs, such as HMGB1, ATP, heat‐shock protein (HSP) and hyaluronan, that could bind to PRRs and activate the immune system. Radiation‐induced DAMP release could explain the radiation‐triggered inflammation.^27^, ^28^, ^29^

HMGB1 is one of the most important DAMP molecules. Some reports have shown that radiation causes HMGB1 release and serves as an extracellular immunoregulator. Some researchers have observed the release of HMGB1 after irradiation and that blocking its release can alleviate RILI,^30^, ^31^ but no information about the involvement of its receptors in the development of RILI has been reported. In our research, we found that whole‐thorax irradiation promoted HMGB1 release into the plasma and increased mRNA expression. HMGB1 protein in the lung tissue changed slightly at the latent stage but decreased significantly at the inflammation stage. At 2 weeks, when the direct effect of irradiation on alveolar epithelial cells was the main effect, HMGB1 release from cells decreased its concentration in lung tissue, while also causing the subsequent synthesis of HMGB1, which was reflected by an increase in mRNA levels. When these reactions reached onto a balance, the expression of HMGB1 in lung tissue remained stable but it still consistently increased the level of HMGB1 in plasma after radiation. At 12 weeks, which is the subsequent chronic inflammation stage, a number of immunocytes infiltrated the pulmonary interstitial tissue, synthesized and secreted HMGB1 under cytokine stimulation. Notably, the secretion exceeded the synthesis, so the HMGB1 level in the lung tissue was lower than that in the control group. Thus, HMGB1 in the lung tissue reflected the dynamic balance between the release and synthesis of HMGB1.

Glycyrrhizin, a natural inhibitor of HMGB1, blocked the release of HMGB1, TNF‐α, IL‐1β and IL‐6 after whole lung irradiation. It also decreased the infiltrating immunocytes in the BALF and lung tissue, which indicated that HMGB1 was an important mediator and a potential therapeutic target of RILI.

RAGE and TLR4 are the receptors to which HMGB1 binds directly. RAGE is moderately expressed in many cell types but highly expressed in alveolar epithelial cells, while TLR4 is expressed mainly in immunocytes.^12^ HMGB1 can regulate the release of TNF‐α, IL‐8, IL‐10 and MCP‐1 via RAGEs in human normal epithelial cells and promote the synthesis of IL‐1β and IL‐18 by the RAGE/NF‐κB pathway in macrophages.^32^, ^33^ HMGB1 also impairs the function of the airway epithelial barrier by activating the RAGE/ERK pathway.^34^ On macrophages, TLR4 is the key receptor for HMGB1, promoting the synthesis and release of TNF‐α.^35^ HMGB1 also regulates T‐cell differentiation via TLR4, RAGE and TLR2 in an asthmatic mouse model.^36^ Both the HMGB1/RAGE and HMGB1/TLR4 pathways participate in sterile inflammation after lung ischaemia‐reperfusion injury.^37^, ^38^ Moreover, both TLR4 and RAGE participate in HMGB1‐mediated lung diseases, although reports on their effects in RILI are scarce. Our research found that there is an early HMGB1 release peak at 2 weeks after irradiation, but the expression of RAGE and TLR4 showed slight changes during the latent stage of RILI. With the progression of disease, the expression of RAGE decreases with alveolar epithelial cell depletion, while TLR4 increases during the acute inflammation stage because of immunocyte infiltration. This finding indicates that the HMGB1/TLR4 axis is the main pathway that mediates RILI.

To exclude the effect of cell numbers at different stages in vivo, we mimicked the process of RILI in vitro. As our research showed, macrophages and lymphocytes are the main infiltrating cells in the inflammation stage. We used RAW 264.7 cells as the infiltrating cells in the alveolar interstitium and MLE‐12 cells as the alveolar epithelial cells. To further illuminate the effect of HMGB1, we examined the supernatants of irradiated MLE‐12 cells and found that the level of HMGB1 was increased at 48 hours but not at 24 hours post‐irradiation. The transwell results showed that migration increased significantly after co‐culture for 48 hours only when HMGB1 was released. To confirm that this chemotaxis is mediated by HMGB1, we added HMGB1 to the lower chamber of the transwell system and found that migrated cells were more obvious than the control cells. This migration can also be inhibited by GL, and HMGB1 can rescue the migration inhibited by GL. Interestingly, radiation did not alter the expression of RAGE and TLR4 on MLE‐12 cells directly. However, replacement of the medium of RAW 264.7 cells with the supernatants of irradiated MLE‐12 after 48 hours, which contained HMGB1, significantly increased the expression of TLR4 in RAW 264.7 cells. Glycyrrhizin can block the migration and inhibit the expression of TLR4 in immunocytes in vivo and in vitro. These results demonstrated that HMGB1 recruits immunocytes to the damaged sites in the lung tissue and mediates inflammation via TLR4 in immunocytes during the progression of RILI.

In contrast to the decrease in RAGE in the lung tissue after irradiation, the level of sRAGE in plasma remained high because of the continuous damage to alveolar epithelial cells in RILI. Soluble RAGE is the C‐terminal splice variant of RAGE that is shed from the plasma membrane via proteolytic cleavage by matrix metalloproteinases. It can be secreted into plasma when damage occurs, so it reflects the severity of cell injury to some extent. As RAGE is highly expressed in alveolar epithelial cells, the plasma level of sRAGE is considered a biomarker for ARDS, ventilation and ventilator‐associated pneumonia and COPD.^39^, ^40^, ^41^ Our research also demonstrated an increase in sRAGE in plasma in accordance with the severity of inflammation even before obvious inflammation occurred; thus, sRAGE could be a biomarker for RILI.

Intracellular transcription factors downstream of HMGB1/TLR4 that mediate sterile inflammation include JNK, ERK1/2 and NF‐κB, etc.^42^, ^43^, ^44^ Our research also examined the downstream effects of HMGB1/TLR4 signals in vivo and vitro and found that the levels of phosphorylated JNK, EKR1/2 and NF‐κB were all increased in lung tissue during the inflammation stage and in RAW 264.7 cells after culture with conditioned medium containing HMGB1.

Glycyrrhizin, an inhibitor of HMGB1, can bind HMGB1 directly without interfering with other chemokines.^15^ GL has many biological effects, such as antiviral, anti‐inflammatory, antitumoral, antioxidant and hepatoprotective effects.^45^ GL can protect against damage caused by drugs, sepsis, ischaemia‐reperfusion and even radiation.^18^, ^46^, ^47^ Our study further confirmed the protective effects of GL against RILI by inhibiting radiation‐induced HMGB1 release and the HMGB1/TLR4 signalling pathway.

In conclusion, our research indicated that HMGB1 is a vital mediator of acute RILI and that its inhibitor GL can mitigate RILI via the HMGB1/TLR4 signalling pathway. The radioprotective effect of GL deserves further investigation and has potential clinical translational value.

## CONFLICT OF INTEREST

The authors declare that they have no competing interests.

## **AUTHORS**'** CONTRIBUTION**

Jia‐Yi Chen and Lei Zheng conceived and designed the research. Lei Zheng conducted most of the experiments, analysed the data and wrote the main part of the paper. All authors participated in parts of the research.

## Data Availability

All data included in this study are available upon request by contact with the corresponding author.
